# Impact of HIV‐infection on human somatosensory processing, spontaneous cortical activity, and cortical thickness: A multimodal neuroimaging approach

**DOI:** 10.1002/hbm.25408

**Published:** 2021-03-18

**Authors:** Chloe C. Casagrande, Brandon J. Lew, Brittany K. Taylor, Mikki Schantell, Jennifer O'Neill, Pamela E. May, Susan Swindells, Tony W. Wilson

**Affiliations:** ^1^ Boys Town National Research Hospital Institute for Human Neuroscience Boys Town Nebraska USA; ^2^ College of Medicine University of Nebraska Medical Center (UNMC) Omaha Nebraska USA; ^3^ Department of Internal Medicine, Division of Infectious Diseases University of Nebraska Medical Center (UNMC) Omaha Nebraska USA; ^4^ Department of Neurological Sciences University of Nebraska Medical Center (UNMC) Omaha Nebraska USA

**Keywords:** gamma oscillations, magnetoencephalography, MRI, somatosensory gating, voxel‐based morphometry

## Abstract

HIV‐infection has been associated with widespread alterations in brain structure and function, although few studies have examined whether such aberrations are co‐localized and the degree to which clinical and cognitive metrics are related. We examine this question in the somatosensory system using high‐resolution structural MRI (sMRI) and magnetoencephalographic (MEG) imaging of neural oscillatory activity. Forty‐four participants with HIV (PWH) and 55 demographically‐matched uninfected controls completed a paired‐pulse somatosensory stimulation paradigm during MEG and underwent 3T sMRI. MEG data were transformed into the time‐frequency domain; significant sensor level responses were imaged using a beamformer. Virtual sensor time series were derived from the peak responses. These data were used to compute response amplitude, sensory gating metrics, and spontaneous cortical activity power. The T1‐weighted sMRI data were processed using morphological methods to derive cortical thickness values across the brain. From these, the cortical thickness of the tissue coinciding with the peak response was estimated. Our findings indicated both PWH and control exhibit somatosensory gating, and that spontaneous cortical activity was significantly stronger in PWH within the left postcentral gyrus. Interestingly, within the same tissue, PWH also had significantly reduced cortical thickness relative to controls. Follow‐up analyses indicated that the reduction in cortical thickness was significantly correlated with CD4 nadir and mediated the relationship between HIV and spontaneous cortical activity within the left postcentral gyrus. These data indicate that PWH have abnormally strong spontaneous cortical activity in the left postcentral gyrus and such elevated activity is driven by locally reduced cortical gray matter thickness.

## Graphical abstract

Forty‐four participants with HIV (PWH) and 55 demographically‐matched uninfected controls completed a paired‐pulse somatosensory stimulation paradigm during MEG and underwent 3T sMRI. These data were used to compute response amplitude, sensory gating metrics, and spontaneous cortical activity power. Our findings indicated both PWH and controls exhibit somatosensory gating, and that spontaneous cortical activity was significantly stronger in PWH within the left postcentral gyrus. Interestingly, within the same tissue, PWH also had significantly reduced cortical thickness relative to controls.

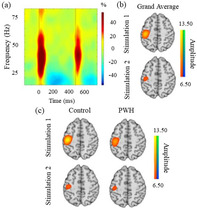

## 1 INTRODUCTION

Over the past 20 years, HIV disease has evolved from a terminal illness to a chronic yet manageable condition with a life expectancy that now approaches that of seronegative persons. However, despite these advances, participants with HIV (PWH) remain at a significantly elevated risk of developing cognitive impairment due to their HIV‐infection, with current data indicating that 35–70% of all PWH develop at least a mild form of HIV‐associated neurocognitive disorder (HAND; Heaton et al., 2010; Heaton et al., 2011; Gannon, Khan, & Kolson, 2011; Simioni et al., 2010; Grant et al., 2014; Sacktor et al., 2002). This persistence is partially driven by the emergence of age‐related cognitive decline in PWH due to the increased longevity of this population, although many other factors also contribute, and the precise mechanisms remain poorly understood.

A growing body of studies have employed neuroimaging techniques to understand the effects of HIV‐infection on the brain (Ances et al., 2009; Ances et al., 2010; Ances, Vaida, Ellis, & Buxton, 2011; Chang et al., 2004; Chang, Yakupov, Nakama, Stokes, & Ernst, 2008; Ernst, Chang, Jovicich, Ames, & Arnold, 2002; Spooner et al., 2018; Wiesman et al., 2018; Wilson, Fox, et al., 2013; Wilson, Heinrichs‐Graham, et al., 2013, 2016; Wilson, et al., 2017, 2019). These studies have shown that HIV‐infection has an extensive effect on both brain structure (Klunder et al., 2008; Sanford et al., 2017; Sanford, Fellows, Ances, & Collins, 2018; Wilson et al., 2015) and function (Simioni et al., 2009; Spooner et al., 2018; Spooner et al., 2020), although the degree to which such structural abnormalities directly translate into functional brain aberrations is unclear. For example, Sanford et al. found reduced volumes in the thalamus and brainstem and cortical thinning in areas such as the primary motor cortex, primary somatosensory cortex, orbitofrontal cortex, cingulate cortex, as well as other frontal and temporal areas in PWH (Sanford et al., 2017, 2018). However, like many neuroimaging studies of HIV, Sanford and colleagues utilized a unimodal neuroimaging approach that focused on structural outcomes, without any direct measure of brain function. Of the few neuroHIV studies that have measured both brain structure and function, Wilson et al. (2015) found reduced gray matter volumes in PWH relative to their uninfected peers in the postcentral gyrus, parahippocampal gyrus, bilateral lingual gyri, and reduced cerebellar volume in crus I, consistent with other findings (Klunder et al., 2008; Wilson et al., 2015). Critically, they found that at least some of the structural findings were accompanied by functional deficits in the same tissue. However, importantly, the sample size in Wilson et al. (2015) was rather limited (17 participants per group), the participants were older (>55 years‐old on average), and the authors did not find any direct link between the structural and functional deficits besides the fact that they occurred in the same tissue. Thus, the relationship between such deficits has yet to be elucidated.

Previous studies have shown that PWH exhibit functional alterations in somatosensory processing, sensory gating, and inhibitory functioning (Spooner et al., 2018; Spooner, Wiesman, et al., 2020), but the degree to which these alterations are attributable to aberrations in brain structure is unknown. Sensory gating is a neurophysiological phenomenon whereby the neural response to the second stimulus in an identical pair is dramatically reduced relative to the first stimulus. This attenuation is thought to reflect the brain's capacity to filter out less relevant sensory information to preserve cognitive resources for more behaviorally relevant stimuli (Cromwell, Mears, Wan, & Boutros, 2008). Sensory gating has been generally understood as a bottom‐up, pre‐attentive process, and numerous studies of somatosensory gating have shown robust oscillations stretching into the high gamma range (i.e., 75 Hz) (Cheng et al., 2016; Kurz, Wiesman, Coolidge, & Wilson, 2018; Spooner et al., 2018; Proskovec, Spooner, Wiesman, & Wilson, 2020; Wiesman et al., 2017). However, of note, a recent study has suggested that attention may modulate somatosensory gating, at least in healthy populations (Wiesman & Wilson, 2020).

In the current study, we utilized magnetoencephalography (MEG) and a paired‐pulse electrical stimulation paradigm to derive functional measures of somatosensory processing, gating, and local spontaneous neural activity, and combined these with computational neuroanatomical analysis of structural MRI data. Our primary goals were to determine whether *functional* brain aberrations observed in PWH were accompanied by *structural* deviations in the same tissue, and to identify the nature of these structural/functional links. In addition, we examined the relationship between key HIV clinical metrics such as disease duration and CD4 nadir and structural and functional brain measures. Our primary hypotheses were that both groups would exhibit sensory gating, but that PWH would have stronger spontaneous cortical activity relative to controls in the left postcentral gyrus, in agreement with prior work (Spooner et al., 2018), and that this increase in spontaneous activity would be mediated by decreased cortical thickness in the same tissue.

## 2 METHODS

### 2.1 Participants

Adult participants (age range: 27–60 years) were drawn from a large ongoing MEG study of healthy and pathological aging (R01‐MH103220). Participants were selected based on their completion of a paired‐pulse MEG paradigm, 3 T structural MRI, and demographics. The exclusionary criteria for this study included any medical illness that affects CNS function (other than HIV), neurological disorders (other than HIV‐associated neurocognitive disorder), psychiatric disease, a history of head trauma, and current substance abuse. All PWH were currently receiving effective combination antiretroviral therapy (cART) and had undetectable viremia at the time of the study. Viral suppression was determined as <50 copies/ml. The group included 109 participants, including 63 uninfected control participants and 46 PWH who were demographically matched based upon ethnicity, age, sex, handedness, and educational levels. All participants provided informed consent consistent with the requirements of the Institutional Review Board of the University of Nebraska Medical Center.

### 2.2 Neuropsychological testing

A neuropsychological battery that assessed multiple domains including attention, fine motor, working memory, language, speed of processing, verbal learning and memory, and executive functioning was completed by each participant. The raw scores from each assessment were then converted into demographically‐adjusted z‐scores (Heaton, Miller, Taylor, & Grant, 2004) and, along with activities of daily living, were used to assess PWH for the presence of HAND. Of note, this battery was consistent with the Frascati consensus (Antinori et al., 2007). None of the uninfected adults were impaired on the neuropsychological assessments or activities of daily living.

### 2.3 Experimental paradigm

Participants were seated with their head within the MEG helmet, as 80 paired‐pulse trials of cutaneous electrical stimulation were applied to their right median nerve using an inter‐stimulation interval (ISI) of 500 ms. An ISI of 500 ms was chosen based on extensive data from previous studies (Arpin, Davies, & Kurz, 2016; Kurz et al., 2018; Spooner, Eastman, Wiesman, & Wilson, 2020; Wiesman et al., 2016). Each pulse consisted of a 0.2 ms square wave, with an amplitude of 10% above the motor threshold. The inter‐pair interval (IPI) varied between 4,500 and 4,800 ms. This paradigm is described in detail and extensively tested in Spooner et al. (2020a).

### 2.4 MEG data acquisition and structural MRI coregistration

Our MEG data acquisition methods are described in detail elsewhere (Wiesman & Wilson, 2020). Briefly, using an acquisition passband of 0.1–330 Hz, neuromagnetic responses were continuously sampled at 1 kHz using a MEG system equipped with 204 planar gradiometers and 102 magnetometers (306 total magnetic sensors; MEGIN, Helsinki, Finland). The resulting MEG data were corrected for head motion, noise reduced (Taulu & Simola, 2006), and coregistered with individual T1‐weighted sMRI data before source space analyses. Following beamformer analysis, each participant's structural and functional images were transformed into standardized space and spatially resampled.

### 2.5 MEG preprocessing, time‐frequency transformation, and sensor‐level statistics

Eye‐blink and cardiac artifacts were removed from the MEG time series using signal‐space projection (SSP; Uusitalo & Ilmoniemi, 1997). The time series was divided into epochs of 3,700 ms duration (−800 to 2,900 ms). This epoch included 400 ms baseline window shifted away from stimulus onset (stimulation 1 onset = 0 ms) from −700 to −300 ms.

Of note, we shifted the baseline window away from the time period immediately before stimulus onset to circumvent any possible contamination by anticipatory responses; however, no such anticipatory responses were detected in our final analyses. For artifact rejection, a fixed threshold method supplemented with visual inspection was used, resulting in an average of 74 trials per participant being retained for source analysis. The mean number of accepted trials per group did not differ (*p* = .30).

Complex demodulation was used to transform the artifact‐free epochs into the time‐frequency domain (Hoechstetter et al., 2004; Kovach & Gander, 2016; Papp & Ktonas, 1977). The resulting spectral power estimations were averaged over trials (per sensor) and normalized per frequency bin using the respective bin's mean power during the −700 to −300 ms baseline period. To derive the time‐frequency windows used for source reconstruction, a two‐stage, data‐driven statistical analysis was conducted on the sensor‐level spectrograms across all participants and gradiometers. This statistical approach is extensively described in our prior work (Spooner, Eastman, et al., 2020; Wiesman & Wilson, 2020). Briefly, paired‐sample t‐tests against baseline were conducted on each pixel in the spectrogram (per sensor), with the output being thresholded at *p* < .05. These potentially significant time‐frequency bins were then evaluated using clustered‐based nonparametric permutation testing (10,000 permutations per comparison; Ernst, 2004; Maris & Oostenveld, 2007), and those bins remaining significant in the permutation analysis were selected for beamforming (see below).

### 2.6 MEG Beamformer imaging and source space analyses

The dynamic imaging of coherent sources (DICS) beamformer was used to image the time‐frequency bins identified in the sensor‐level analysis (Van Veen, Drongelen, Yuchtman, & Suzuki, 1997; Gross et al., 2001). Essentially, using the DICS approach, we computed normalized source power for the selected time‐frequency periods (see *Results*) per participant at 4.0 × 4.0 × 4.0 mm resolution. The resulting images were averaged across both stimulations and all participants to assess the anatomical basis of the oscillatory activity identified through the sensor‐level analysis. Using the peak voxel coordinates derived from this grand‐averaged image, virtual sensors (i.e., voxel time series) were extracted from each participant's data by applying the sensor weighting matrix to the preprocessed signal vector. Note that we extracted virtual sensor data from each participant using the peak coordinate from the grand‐averaged image, and then estimated the envelope of the time series for the spectral band identified in the sensor level analysis. Using these envelopes, we computed the relative (baseline‐corrected) and absolute time series for each participant. This data processing pipeline is extensively described elsewhere (Spooner, Eastman, et al., 2020).

### 2.7 Structural MRI morphometry

A Philips Achieva 3 T scanner equipped with an eight‐channel head coil was used to acquire structural T1‐weighted MRI data. We used a 3D fast field echo sequence with these parameters: TR: 8.09 ms; TE: 3.7 ms; the field of view: 24 cm; matrix: 256 × 256; slice thickness: 1 mm with no gap; in‐plane resolution: 0.9375 × 0.9375 mm; sense factor: 1.5. The details of our MRI data processing pipeline are available (Proskovec et al., 2020). Briefly, we used the Computational Anatomy Toolbox (CAT12 v12.6; Gaser & Dahnke, 2016) within SPM12 to conduct standard surface‐based morphometry (SBM) on the high‐resolution structural data. Fundamentally, the SBM pipeline within the CAT12 toolbox is an automated approach that employs a projection‐based thickness (PBT) approach to estimate local cortical thickness and reconstruct the central surface (Dahnke, Yotter, & Gaser, 2013). The PBT approach accounts for partial volume correction, sulcal blurring, and sulcal asymmetries without sulcal reconstruction. To rectify defects in topology, correction based upon spherical harmonics was used (Yotter, Dahnke, Thompson, & Gaser, 2011), and the resulting cortical surface mesh was reparametrized into a common coordinate system. This was accomplished by an algorithm that reduces area distortion (Yotter, Thompson, & Gaser, 2011). The resulting maps were finally resampled and smoothed using a 15 mm FWHM Gaussian kernel.

Our CAT12 pipeline included a noise reduction step (Manjón, Coupé, Martí‐Bonmatí, Collins, & Robles, 2010), the Markov Random Field approach (Rajapakse, Giedd, & Rapoport, 1997), and affine registration and local intensity transformation to the bias‐corrected images. An adaptive maximum a posteriori technique (Ashburner & Friston, 2005) was then used to segment the images, with partial volume estimation based on a simplified mixed model (Tohka, Zijdenbos, & Evans, 2004). Once the MRI data were fully processed and in MNI space, a mask was constructed for the cortical surface mesh using the same peak voxel coordinates as the MEG virtual sensor extraction. Briefly, using the WFU Pick atlas (version 3.0; Maldjian, Laurienti, Kraft, & Burdette, 2003; Maldjian, Laurienti, & Burdette, 2004), an 8 mm sphere centered on the MEG peak voxel was generated, and this was spatially resampled to 1 mm to align with the structural data. Using the transform provided in CAT12, this normalized volume mask was then transformed into the surface template space, which allowed cortical thickness values within the mask to be extracted per participant. For clarity, the final values reflect the mean cortical thickness across the mask. This masking procedure is also described in Proskovec, Spooner, et al., 2020. Finally, to enhance rigor, we derived mean cortical thickness using spheres of varying sizes (8, 9, and 10 mm) centered on the same peak MEG coordinates, and across all sizes, our statistically significant findings remained.

### 2.8 Statistics

We used mixed‐model ANOVAs to evaluate group differences in somatosensory processing and gating and t‐tests to evaluate group differences in spontaneous activity and cortical thickness. To identify relationships between brain structure and function and determine how these are modulated by HIV‐infection, we used structural equation modeling. Statistical significance of indirect effects was determined using bias‐corrected bootstrapped confidence intervals (Efron & Tibshirani, 1986; Fritz & MacKinnon, 2007) around all indirect effects using 1,000 bootstrapped samples. Note that because of the use of confidence intervals to determine significance, exact *p*‐values are not available for indirect effects. As a follow‐up to these analyses, we examined whether any clinical measures of HIV (i.e., CD4 nadir) could explain the noted effects of HIV group membership on brain structure and function. These models were tested only in PWH. We utilized full information maximum likelihood (FIML) to estimate any missing data in all models; analyses were conducted using Mplus version 8.1.

## 3 RESULTS

Of the 109 participants 10 participants were excluded due to artifacts in their MEG and/or MRI data. The remaining 99 participants, 55 controls and 44 PWH, did not statistically differ in age, sex, education, ethnicity, or other demographic variables (see Table 1). Fifteen of the 44 PWH met the criteria for HAND according to the Frascati guidelines. For the PWH, time since diagnosis, current CD4, and CD4 nadir were recorded at the time of enrollment.

**TABLE 1:** Participant demographics

	Control (*n* = 55)	PWH (*n* = 44)	Significance
Age (years)	42.5 ± 10.6	45.9 ± 10.0	*p* = .358
Sex	32 M, 23 F	26 M, 18 F	*p* = .991
Handedness	51 R, 4 L	41 R, 3 L	*p* = .931
Weight (kg)	91.9 ± 23.8	85.1 ± 23.5	*p* = .212
Education (years)	16.8 ± 2.34	14.5 ± 2.22	*p* = .500
Time on cART (years)	–	9.12 ± 6.01	
Time since HIV diagnosis (years)	–	10.8 ± 6.48	
CD4 nadir (cells/μl)	–	209 ± 159	
Current CD4 (cells/μl)	–	792 ± 460	

*Note:* Values displayed are mean ± *SD*.

Abbreviations: cART, combination antiretroviral therapy; F, female; L, left; M, male; R, right.

### 3.1 MEG sensor‐level analysis

**FIGURE 1:**
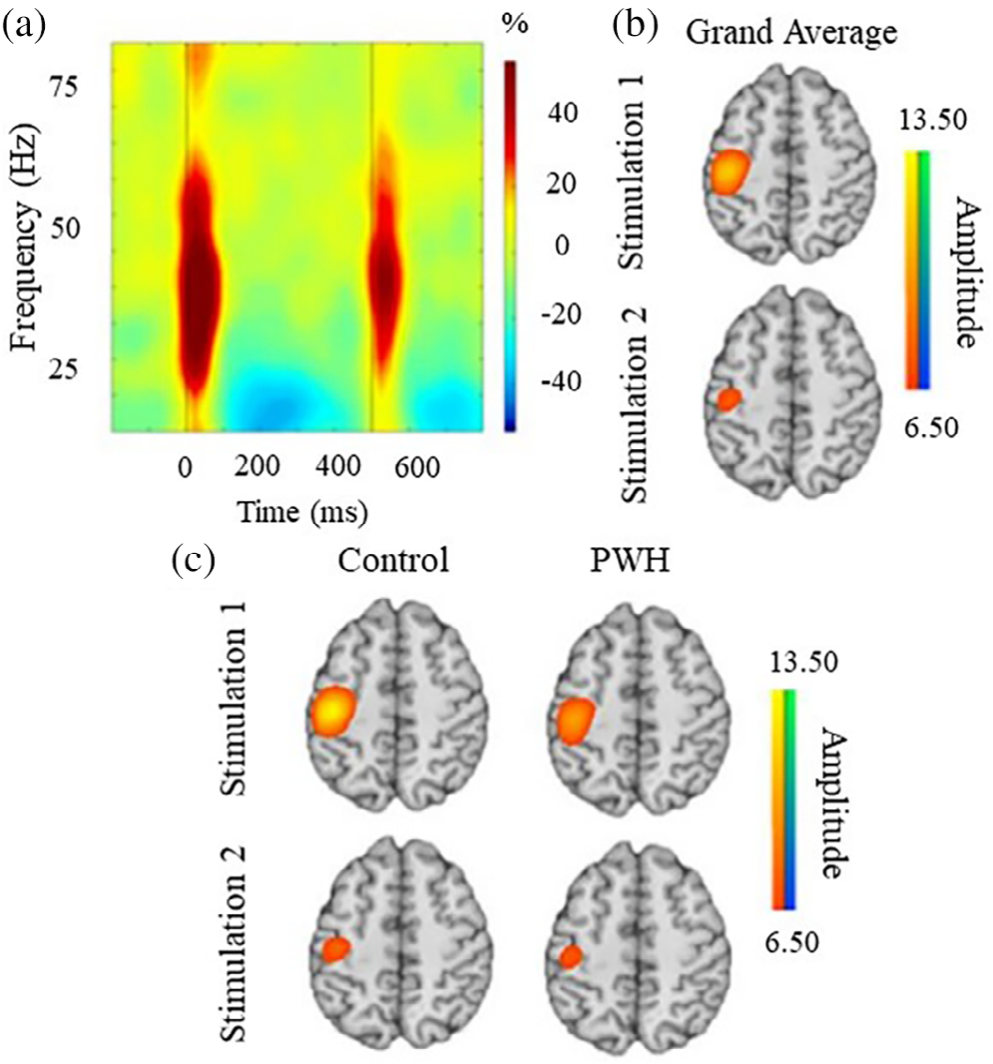
Neural responses to paired‐pulse somatosensory stimulations to the right median nerve. (a) Time frequency spectrogram depicting broadband somatosensory responses from a representative MEG sensor near the left somatosensory cortex. The x‐axis denotes time (ms) while the y‐axis denotes the frequency (Hz). The stimulation onsets occurred at 0 ms for stimulation 1 and 500 ms for stimulation 2. The color bar to the right of the spectrogram indicates the color scale in percent change from baseline (−700 to −300 ms) units. (b) Grand‐averaged beamformer images collapsed across both groups for the first and second stimulation. (c) Group‐averaged beamformer images for stimulation 1 responses (top row) and stimulation 2 (bottom row). Both groups exhibited a strong increase in the 20–75 Hz spectral window during the 50 ms following each stimulation. Note that the color scale bar to the right of each group of images reflects the threshold in positive (warmer colors) and negative (cooler colors) pseudo‐t values. Across groups, the brain area exhibiting the largest response was virtually identical and was centered on the contralateral hand region of the somatosensory cortex. Note that the peak voxel in the grand‐averaged image was used for virtual sensor extraction and the additional analysis described in the methods

Robust broadband synchronizations spanning 10–90 Hz were observed in several MEG sensors near the sensorimotor and parietal regions during the 100 ms directly following the onset of electrical stimulation (*p* < .001, corrected). These responses, especially those in gamma frequencies, were considerably stronger in the 50 ms immediately following each electrical stimulation (Figure 1a). Thus, we focused our beamformer analyses on these 50 ms time windows (i.e., 0–50 ms and 500–550 ms) following each stimulation and the 20–75 Hz range. Our main analyses started at 20 Hz as this was the lowest frequency that we could precisely resolve using a 50 ms time interval. We stopped at 75 Hz on the high end as the relative power of the neuronal responses decreased sharply thereafter, particularly in response to the second stimulus.

### 3.2 HIV‐related alterations in somatosensory cortical structure and function

Beamformer output images indicated robust responses in the contralateral somatosensory hand region of the postcentral gyrus following each stimulation (Figure 1b). The peak locations of the responses to the first and second stimulations were virtually identical in the contralateral postcentral gyrus, and these locations were highly similar across the two groups (Figure 1c). As described in the *Methods* section, the beamformer images were grand averaged across all participants and stimulations, and then virtual sensor data were extracted in each participant from the peak voxel in the grand‐averaged image. The envelope of the resulting time series was then computed for the 20–75 Hz frequency range.

**FIGURE 2:**
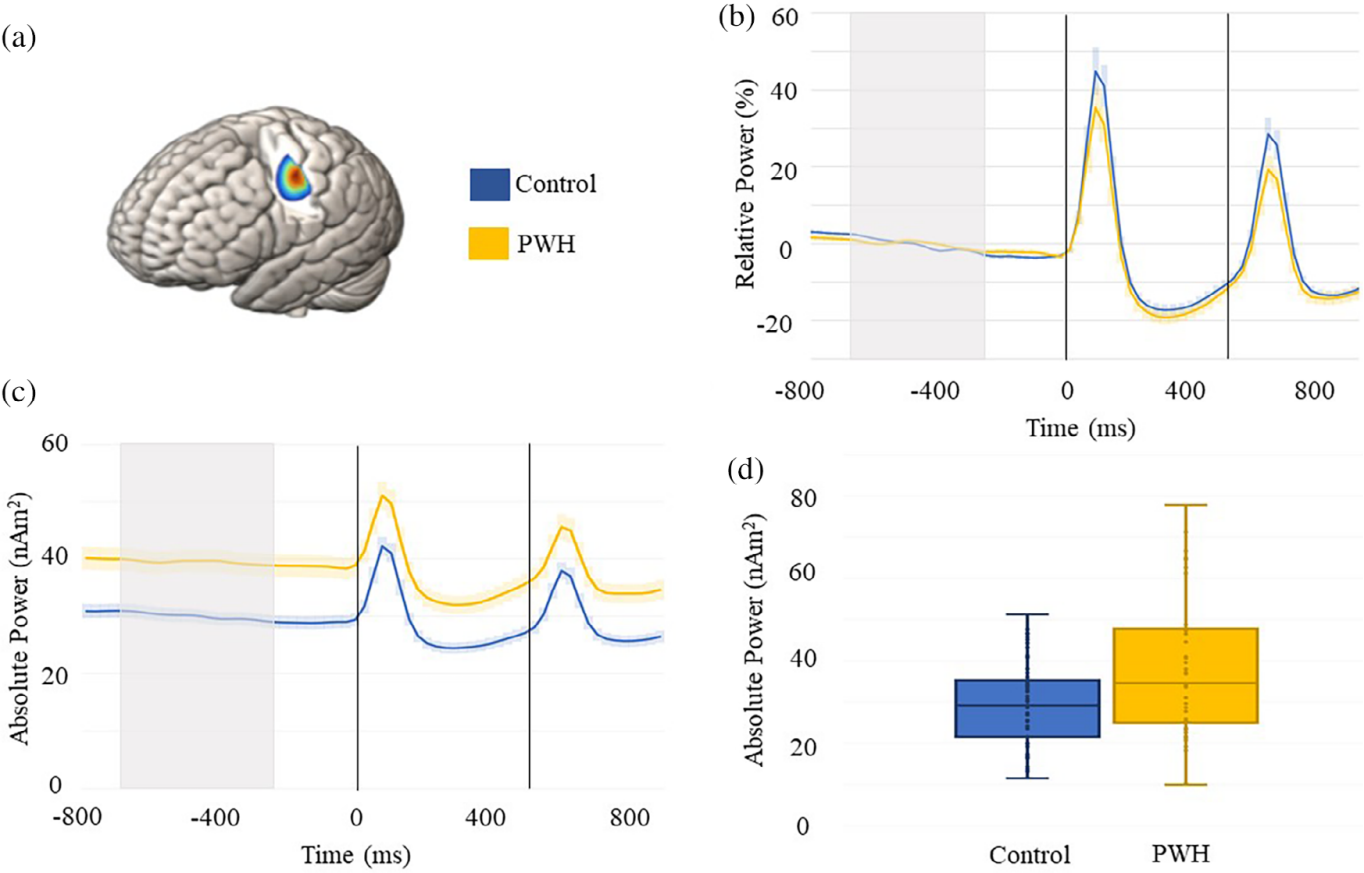
MEG time series of somatosensory processing and spontaneous activity. (a) Grand‐averaged beamformer image for peak voxel virtual sensor extraction. (b) Relative time series in PWH and controls showing the significant sensory gating effect (i.e., stronger response to stimulation 1 compared with stimulation 2) across both groups. The dashed lines indicate the onset of each stimulation, with controls shown in blue and PWH in yellow. The shaded area around each time series reflects the *SEM*. (c) Absolute voxel time series showing the elevated spontaneous activity during the baseline period in PWH compared with controls. The gray shaded area reflects the baseline period (−700 to −300 ms) used to estimate the average. (d) Box plots showing the spontaneous power data in each group. The x indicates the mean, with the horizontal lines marking the median and first and third quartiles, and the dots indicate individual data points

To examine group differences in sensory processing, we computed two mixed‐model 2 × 2 analysis of variances (ANOVAs) (stimulation‐by‐group). Participants with responses outside of ±2.5 *SD* were excluded from these analyses (*n* = 2). The first examined response amplitude and the second tested peak frequency. The analysis of response amplitude indicated a main effect of stimulation, such that the response to stimulation 2 was weaker than that to stimulation 1 across all participants (*F*[1,96] = 45.65, *p* < .001). In other words, there was a significant somatosensory gating effect across all participants (Figure 2b). In contrast, neither the main effect of the group (*p* = .159) nor the interaction were significant (*p* = .776). As per peak frequency, the main effect of stimulation was significant (*F*[1,96] = 4.19, *p* < .05), indicating that the peak frequency of the response to the second stimulation (mean = 49.7, *SD* = 14.9) was higher than that to the first stimulation (mean = 45.0, *SD* = 14.9). Similar to the amplitude findings, neither the main effect of the group (*p* = .385) nor the interaction were significant (*p* = .386).

Next, we examined the strength of spontaneous neural activity during the baseline period by computing the mean amplitude from −700 to −300 ms using the absolute power time series (i.e., not baseline corrected). Independent sample t‐tests indicated significantly stronger spontaneous power in the PWH relative to controls (*p* = .001; Figure 2c,d). We further explored the extent to which these group‐level differences in neural activity may be explained by group differences in cortical structure, namely cortical thickness within the left postcentral gyrus.

### 3.3 Local cortical thickness mediates the effect of HIV on spontaneous neural activity

**FIGURE 3:**
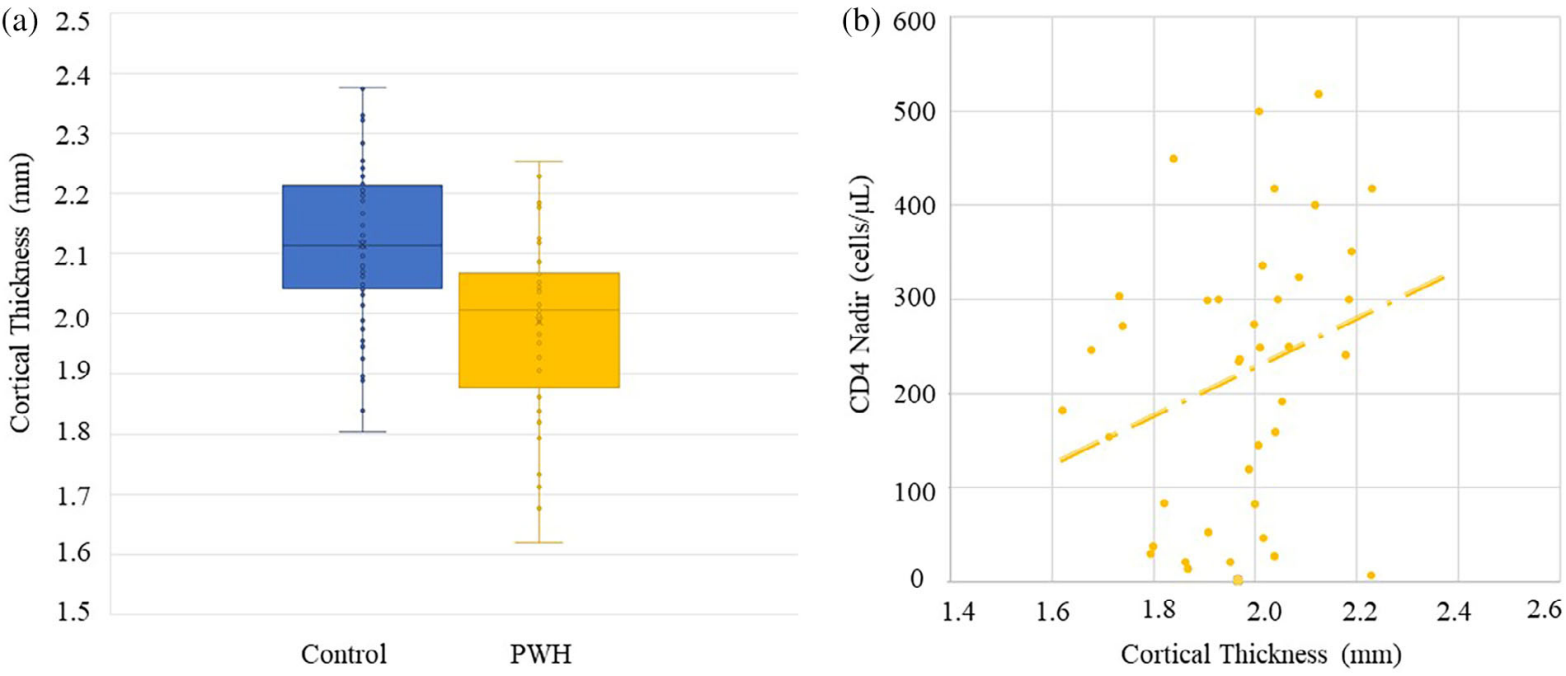
Cortical thickness in left postcentral gyrus and correlations. (a) Box plots showing the cortical thickness results in each group. The x indicates the mean, with the horizontal lines marking the median, and first and third quartiles, and the dots indicate the individual data points. (b) Correlation between cortical thickness and CD4 nadir in PWH group (ρ = 0.326, *p* = .043) indicating that those with lower CD4 nadir also had the thinnest cortex in the left postcentral gyrus

**FIGURE 4:**
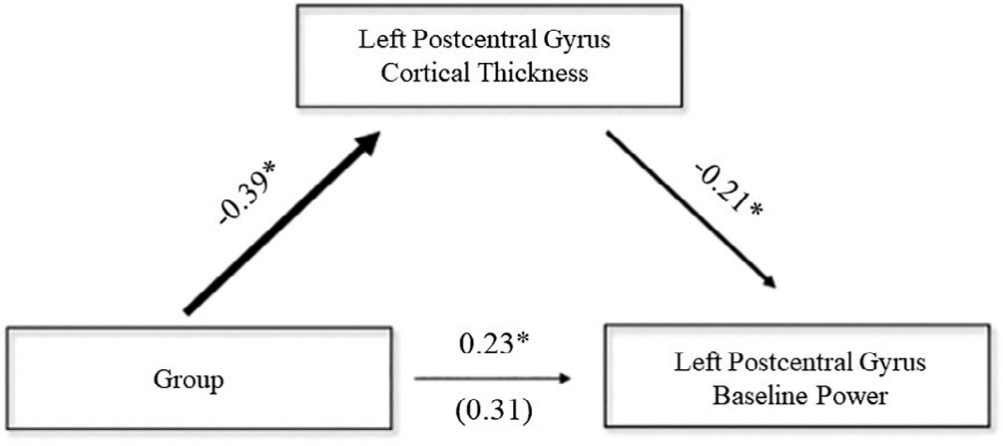
Mediation analysis. The mediation model whereby cortical thickness in the left postcentral gyrus serves as a mediator of the relationships between diagnostic group (PWH or control) and spontaneous power during the baseline period within the same cortical tissue. All reported parameters are standardized coefficients. The value in parentheses is the total effect of the group on spontaneous baseline power. Each effect was statistically significant at the *p* < .05 level, as assessed by bias‐corrected bootstrapped confidence intervals. *p < .05

To evaluate differences in cortical thickness, we computed the mean cortical thickness in each participant within an 8 mm sphere centered on the peak somatosensory response in the left postcentral gyrus, which was derived from the grand‐averaged MEG beamformer images. These mean values were examined using independent sample *t* tests which revealed significant group differences (Figure 3a) such that PWH had significantly reduced cortical thickness relative to controls. Then, to evaluate the extent to which gray matter thickness within the left postcentral gyrus mediated the effects between HIV and spontaneous neural activity, we tested a mediation model in which diagnostic group (i.e., PWH or control) predicted gray matter thickness within the left postcentral gyrus which subsequently predicted spontaneous neural activity within the same brain tissue. As identified in our primary analysis, there was a significant total effect of HIV on spontaneous activity (β = .31, b = 4.44, 95% CI [1.51, 7.30]),

such that PWH tended to have higher resting power relative to controls. Importantly, there was also a significant mediation whereby cortical thickness in the left postcentral gyrus partially explained the relationship between HIV and spontaneous neural activity during the baseline (indirect: β = .080, b = 1.15, 95% CI [.087, 2.65]; Figure 4). Specifically, PWH had significantly reduced postcentral gyrus thickness relative to healthy controls (β = −.39, b = −.062, *p* < .001), and this reduced thickness subsequently predicted stronger spontaneous power during the baseline period (β = −.21, b = −18.59, *p* = .040). Note that the direct effect of HIV on spontaneous activity remained significant (β = .23, b = 3.29, 95% CI [.33, 5.94]). There were no other statistically significant relationships between group membership and brain structure or function.

To follow up these analyses, we tested an exploratory model exclusively in PWH to determine whether indices of disease severity could explain the noted effects of HIV‐infection on cortical thickness and spontaneous power in the left postcentral gyrus. The model was structured such that current CD4 counts, CD4 nadir, and time since HIV diagnosis all served as predictors of spontaneous activity before somatosensory stimulation with gray matter thickness in the left postcentral gyrus modeled as a mediator. Indices of HIV disease were allowed to freely correlate. Postcentral gyrus thickness was significantly predicted by CD4 nadir (β = .31, b = .004, *p* = .04) indicating that individuals with lower CD4 nadir tended to have thinner gray matter in the left postcentral gyrus (Figure 3b). However, there were no other significant associations between markers of HIV disease and brain structure or function, nor were there any significant indirect effects.

## 4 DISCUSSION

The primary goal of this study was to examine the relationship between brain structure and function in the postcentral gyrus of PWH with virologic suppression. To this end, participants underwent paired‐pulse electrical stimulation of the right median nerve during MEG, as well as a 3 T structural MRI session. We hypothesized that PWH would exhibit normal sensory gating but robustly stronger spontaneous baseline power relative to controls in the postcentral gyrus, consistent with findings from Spooner et al. (2018), and that this increased spontaneous neural activity would be mediated by locally reduced cortical thickness in the left postcentral gyrus. Our results indicated that PWH had relatively preserved somatosensory gating, as well as reduced cortical thickness and increased spontaneous neural activity in the brain tissue generating the strongest somatosensory response. Below, we discuss the implications of these findings, with an emphasis on the spontaneous cortical activity, the novelty of the structure/function relationships identified in this study, and the relationships between CD4 nadir and cortical thickness.

One of our principal findings was the increase in spontaneous neural activity in the left postcentral gyrus of PWH compared with controls. Such increased spontaneous neural activity has previously been reported in healthy older relative to younger adults in the primary motor (Heinrichs‐Graham et al., 2018; Heinrichs‐Graham & Wilson, 2016) and somatosensory cortices (Spooner, Wiesman, Proskovec, Heinrichs‐Graham, & Wilson, 2019), and has been linked to altered neural oscillations and impaired behavioral performance in older adults (Heinrichs‐Graham & Wilson, 2016). Such spontaneous activity is also known to increase throughout the day and be strongest in the late afternoon (Wilson, Heinrichs‐Graham, & Becker, 2014) when motor performance is known to be less precise (Carrier & Monk, 2000; Drust, Waterhouse, Atkinson, Edwards, & Reilly, 2005). Thus, given the evidence for accelerated aging in HIV (Gross et al., 2016; Holt, Kraft‐Terry, & Chang, 2012; Horvath & Levine, 2015; Lew et al., 2021; Pathai, Bajillan, Landay, & High, 2013), our finding of robustly increased spontaneous neural activity in PWH is not surprising. In fact, previous studies have shown increased spontaneous alpha and gamma in the occipital cortices of PWH (Wiesman et al., 2018), as well as increases in spontaneous theta and alpha in frontal, parietal, and occipital cortices serving selective attention in PWH relative to demographically‐matched controls (Lew et al., 2018). Of note, the Lew et al. study found a step‐wise increase in spontaneous power insofar as control participants exhibited the lowest spontaneous power in theta and alpha activity, PWH had slightly elevated spontaneous power in these bands, and impaired PWH (persons with HAND) exhibited the highest spontaneous power in the theta and alpha canonical bands. This pattern was observed in several regions of interest providing evidence for the stability of this pattern. The ability to distinguish those with HAND from unimpaired PWH and controls in multiple brain regions was also observed by Wiesman and colleagues (Wiesman, Mills, et al., 2018). Similar findings of elevated spontaneous activity distinguishing those with HAND from unimpaired PWH and controls was also recently reported in the somatosensory cortices (Spooner, Wiesman, et al., 2020). Given these data and the findings from healthy aging, such increases in spontaneous activity almost certainly reflect pathological changes in local circuit function, and studies that have collected behavioral performance metrics in parallel with MEG have indeed supported this (Lew et al., 2018; Wiesman, O'Neill, et al., 2018). Unfortunately, our MEG task did not require behavioral responses and thus we cannot directly comment on whether the increased spontaneous activity would have impacted cognition. Nonetheless, the converging evidence reveals elevated spontaneous activity impairs local circuit function and may lead to a decline in cognitive performance. Given recent evidence that noninvasive electrical brain stimulation can modulate local spontaneous activity in healthy adults (Heinrichs‐Graham, McDermott, Mills, Coolidge, & Wilson, 2017; McDermott et al., 2019; Wiesman, O'Neill, et al., 2018; Wilson, Mcdermott, Mills, Coolidge, & Heinrichs‐Graham, 2018), studies examining the efficacy of such stimulation in PWH could be fruitful.

Our structural findings of reduced cortical thickness in the somatosensory cortex in PWH is consistent with previous structural work (Cardenas et al., 2009; Lew et al., 2021; Wilson et al., 2015). Evidence suggests that even in the cART era, PWH experience progressive gray matter volume loss, as well as abnormally diminished white matter volumes and increased ventricular volumes (Cardenas et al., 2009; Cohen et al., 2010), although cART has unquestionably reduced the severity of volume and cortical thickness reductions in PWH (Cohen et al., 2010; Heindel et al., 1994; Stout et al., 1998; Thompson et al., 2005). An interesting finding in our study was the relationship between CD4 nadir and brain structure in PWH, such that lower CD4 nadir values were associated with greater cortical thinning in the left postcentral gyrus. This finding could indicate that the severity of these neural deficits is at least partially attributable to legacy effects related to the early stages of the infection. Similar findings linking CD4 nadir and neuropsychological function have been widely reported (Cysique & Brew, 2009; Ellis et al., 2011; Jernigan et al., 2011; Robertson et al., 2007; Tozzi et al., 2007).

A key novel finding of this study was the critical link between aberrant thinning in the left postcentral gyrus and the stronger spontaneous cortical activity within the same tissue in PWH. Essentially, our mediation model revealed a fully‐mediated effect whereby HIV‐infection predicted decreased cortical thickness in the left postcentral gyrus, which subsequently predicted increased baseline power in the same brain tissue. This novel structure/function relationship suggests that the loss of neural tissue is directly related to the HIV‐associated alterations in spontaneous power, which is critical for understanding the neuropathology of HIV. Further, these data fill a key missing piece of the puzzle, as the net impact of structural degeneration on neural function in PWH has remained unclear. Essentially, prior studies have generally examined either brain structure or function, and those that have examined both have either not tested a direct relationship between the findings and/or had too small of a sample for such analyses (i.e., see Wilson et al., 2015). While these findings are compelling, it should be noted that we restricted our analyses to the left postcentral gyrus and cannot comment on wider associations. Future studies should directly test other brain regions for such structure/function relationships in PWH.

Before closing, it is important to acknowledge the limitations of this study. First, our study focused on virally‐suppressed PWH who did not have neurologic or psychiatric illnesses (except HAND), acute concurrent illnesses, or major substance abuse or depression symptoms. Thus, the sample was relatively healthy, and our findings may not generalize to samples that include individuals with high viral loads, depression, or other neurologic or psychiatric conditions. Second, our PWH group included patients with and without cognitive impairment (i.e., HAND) and future studies should examine the impact of HAND directly. We would speculate that the structure/function relationships identified here would not be affected by HAND, but that should be confirmed. Third, as stated previously, our study focused on the left postcentral gyrus, and future studies should use higher‐level tasks to activate widespread brain networks which could then be probed for similar structure/function relationships as observed here. Again, we would speculate that the relationship would hold in other brain regions, but directly testing this would be a strong next step. Fourth, the cross‐sectional nature of the study design limits our understanding of the trajectory of these deficits, and thus future longitudinal studies would be very informative. Finally, we only stimulated the right hand and did not investigate hemispheric asymmetries in brain structure or function. Future studies should extend these findings by stimulating the left hand and evaluating possible hemispheric differences in both brain function (i.e., somatosensory function) and cortical thickness. Despite these limitations, the current study provides critical new data linking aberrant thinning in the somatosensory cortices of PWH to altered spontaneous cortical activity in the same brain tissue.

## CONFLICT OF INTEREST

The authors have no competing financial or other interests.

## AUTHOR CONTRIBUTIONS

Chloe Casagrande: data curation, formal analysis, writing‐ original draft. Brandon Lew: data curation, formal analysis, writing‐ review, and editing. Brittany Taylor: formal analysis, writing‐ review, and editing. Mikki Schantell: data curation, project administration, writing‐ review, and editing. Jennifer O'Neill: data curation, project administration. Pamela May formal analysis, writing‐ review, and editing. Susan Swindells: conceptualization, funding acquisition, investigation, methodology, writing‐ review, and editing. Tony Wilson: conceptualization, funding acquisition, methodology, writing‐ review, and editing.

## Data Availability

The data that support the findings of this study are available from the corresponding author upon reasonable request.
